# Understanding dual precipitation strengthening in ultra-high strength low carbon steel containing nano-sized copper precipitates and carbides

**DOI:** 10.1186/s40580-017-0110-5

**Published:** 2017-07-03

**Authors:** M. P. Phaniraj, Young-Min Shin, Woo-Sang Jung, Man-Ho Kim, In-Suk Choi

**Affiliations:** 1 High Temperature Energy Materials Research Center, Korea Institute of Science and Technology, Seoul, 136-791 Republic of Korea; 2 Advanced Analysis Center, Korea Institute of Science and Technology, Seoul, 136-791 Republic of Korea

**Keywords:** HSLA steel, Copper, Nano-sized carbides, Interrupted cooling, SANS

## Abstract

Low carbon ferritic steel alloyed with Ti, Mo and Cu was hot rolled and interrupt cooled to produce nano-sized precipitates of copper and (Ti,Mo)C carbides. The steel had a tensile strength of 840 MPa, an increase in yield strength of 380 MPa over that of the plain carbon steel and reasonable ductility. Transmission electron microscopy and small angle neutron scattering were used to characterize size and volume fraction of the precipitates in the steels designed to form only copper precipitates and only (Ti,Mo)C carbides. The individual and combined precipitation strengthening contributions was calculated using the size and volume fraction of precipitates and compared with the measured values.

**Electronic supplementary material:**

The online version of this article (doi:10.1186/s40580-017-0110-5) contains supplementary material, which is available to authorized users.

## Background

While conventional microalloyed ferritic steels typically possess yield strengths in the range 450–550 MPa [1, 2], significant improvements in strengthening with nano-sized precipitates can be achieved by a combination of alloy design and thermomechanical processing followed by controlled cooling. Funakawa et al. [3] reported an increase in yield strength by 300 MPa in the hot rolled and control-cooled low carbon steel containing Ti and Mo in equiatomic concentration. The high strength of the steel was primarily due to the precipitation of nanometer size (Ti,Mo)C carbides (~3 nm). Chen et al. [4] compared the properties of continuously cooled low carbon steels that were microalloyed with Ti, Ti and Mo, and Ti and Nb. They reported that (Ti,Mo)C carbides had finer size and imparted higher hardness to the steel. The (Ti,Mo)C carbides were also thermally stable relative to carbides in Ti and Ti-Nb microalloyed steels.

Copper addition in amounts of 1–2 wt% has also been used for precipitation strengthening in low carbon structural steels [5–9]. Commercially available copper containing low carbon steels such as ASTM A710 or HSLA80, also contain microalloying elements such as Nb and have yield strength between 450–520 MPa [10, 11] in the as-rolled and air-cooled condition. The higher strength of these steels is because of the formation of nanosized copper precipitates in ferrite and grain refinement brought out by microalloy carbides in prior austenite. The strength of copper containing low carbon steels was further increased by microalloying with a combination of carbide forming elements such as V, Nb, Ti and Mo to ~660 MPa. Recently, the present authors reported that the yield strength of 730 MPa can be achieved in low carbon steel alloyed with Cu, Ti and Mo [12]. The increase in strength was achieved by the dual precipitation of nano-sized (Ti,Mo)C carbides and copper precipitates using a two-step interrupted cooling after hot rolling. However, the contribution of Cu and (Ti, Mo) C to precipitation strengthening could not be distinguished because quantification of precipitation hardening is a challenging subject as it demands combined knowledge of precipitation strengthening mechanism and reliable measurement of precipitates size and volume fraction. Particularly, the copper precipitates could not be detected using transmission electron microscopy (TEM) and scanning electron microscopy (SEM) because their small size (~3 nm) and poor diffraction contrast when they are fully coherent with matrix [13, 14].

Small angle neutron scattering (SANS), which has merit in the averaged value since it measures larger sample size, has typically been used to determine size and volume fraction of nanometer sized copper precipitates in Fe–Cu alloys [13, 15–19]. Wiskel et al. [20] quantified precipitate size and distribution in commercial steels, containing different amounts of copper and the carbide forming elements Nb and Ti, using SANS. They reported that the steels contained precipitates with a bimodal size distribution. However, they could not determine if the bimodal distribution came from copper or carbide precipitates alone, or from a mixture of carbide and copper precipitates. In steels forming multiple precipitates of similar size, it is difficult to analyze SANS data. To determine size distribution of copper precipitates in the commercial steel A710 that formed similar sized copper and niobium carbide precipitates Pande et al. [13] prepared the steel without the carbide forming element.

In the present study, we systematically investigate how each nano-precipitation contributes to strengthening in ultra-high strength low carbon ferritic steel containing nano-sized copper precipitates and carbides using SANS in combination with SEM and TEM. Low carbon steel containing copper and Ti-Mo were hot rolled followed by interrupted cooling. TEM and SANS analysis to determine size and volume fraction were carried out on steels that were designed to form only copper and only carbide precipitates. The individual and combined precipitation strengthening contributions were calculated based on strengthening mechanism and compared with measured values.

## Experimental details

The composition of steels is given in Table 1. Four different steels were prepared which are denoted as CMn, TiMo, 1.7 Cu and 1.7 CuTiMo. Compared to our previous study, we increase Cu contents to 1.7% at which the size of Cu precipitates can be detected by using both TEM microscope and SANS. The steels were induction melted, forged and hot rolled into 15 mm thick [12]. The slabs were then heated to 1250 °C and held for 30 min to dissolve any precipitates and then rolled to 75% reduction at 900 °C. After rolling the specimens were first air cooled to 650 °C and held for 5 min, followed by air cooling to 500 °C where it was held for 60 min and then furnace cooled to room temperature. The precipitates viz. (Ti,Mo)C carbide and Cu rich phase are expected to form at 650 °C [3, 4, 12, 21] and 500 °C [12, 22, 23] respectively. Specimens for the tension test were prepared from the rolled plates along the rolling direction according to ASTM standard E08-M with gage length of 25 mm, gage width of 6 mm and thickness of 2 mm. The tests were carried out at the constant crosshead speed of 1 mm/min. The tension test experiments were conducted on two specimens for each composition. The microstructure was characterized using SEM, the 200 kV Tecnai 20 TEM. Focused Ion Beam (FIB) technique was used to make thin foil specimens for characterization of precipitates. The precipitates were also extracted by dissolution in the electrolyte solution consisting of 4% tetramethylammonium chloride, 10% acetone and 86% methyl alcohol.

**Table 1:** Chemical composition of steels (wt%)

Steel	C	Mn	Si	Mo	Ti	Cu	Al	Fe
CMn	0.07	1.47	0.32	–	–	–	0.04	Bal.
TiMo	0.07	1.34	0.32	0.20	0.09	–	0.04	Bal.
1.7 Cu	0.06	1.50	0.32	–	–	1.69	0.04	Bal.
1.7 CuTiMo	0.07	1.54	0.33	0.21	0.12	1.72	0.03	Bal.

SANS experiments were also performed to characterize nano-precipitates in the steels at room temperature at the HANARO Cold Neutron facility [24] which is equipped with a two-dimensional position sensitive detector. Flat samples with the dimensions of 5 × 2.2 × 1.5 mm were placed between the two electric magnetic poles, where the external magnetic field (1.0 T) was applied horizontally, parallel to the sample surface, and perpendicular to the incident neutron beam. The scattering vector range, ~0.004 < *Q*(=4*π* sin*θ*/) < ~0.17° Å^−1^ where 2θ is the scattering angle presented here was collected from two detector distances: 3 and 9 m corresponding to neutron wavelengths of 4.45 and 8.28 Å. The measured intensities were converted to the absolute scale (i.e. coherent macroscopic scattering cross section $d\sum {(Q)} /d \varOmega$) by correcting them with a standard silica sample with known R_g_ and I(0). The scattering cross section consists of the magnetic and nuclear components. In the vertical plane both nuclear and magnetic scattering occur whereas in the horizontal plane only nuclear scattering occurs. The averaged nuclear scattering data within the sector of 10° that is an angle between the magnetic field and the scattering vector was used in the present study for the analysis. The data were analyzed using the macros, provided by National Institute for Standards and Technology, USA [25] for the Igor Pro software. Using the macros in the software the scattering profile was simulated with a geometric model, such as a sphere or an elliptic cylinder, for the precipitate. The simulated profile was then fitted iteratively, by adjusting model parameters, to the measured data to determine precipitate size and distribution. The equations for fitting the data with the geometric models as well as their corresponding references are available in Ref. [26].

## Results and discussion

### Microstructure

**Fig. 1:**
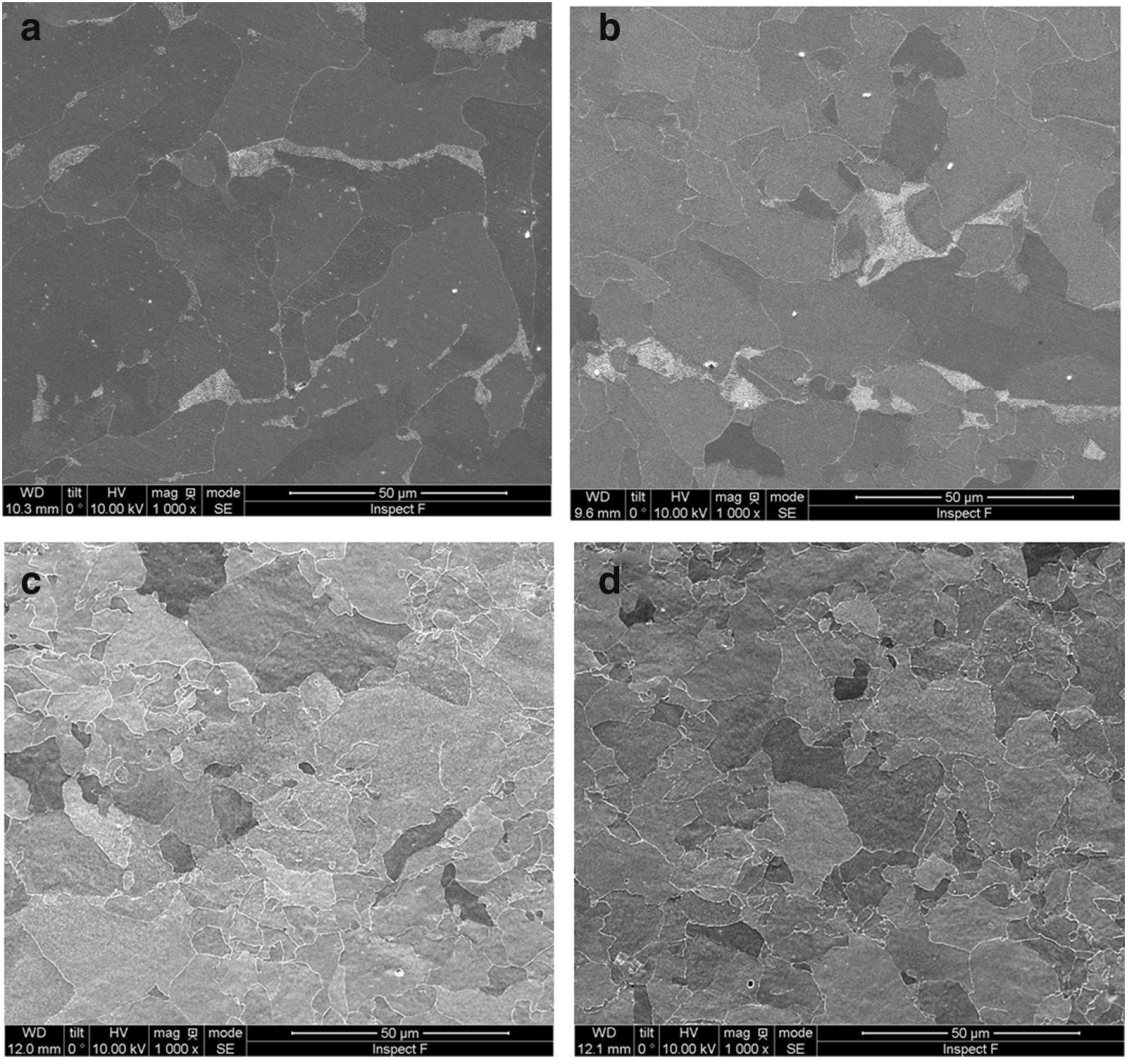
SEM micrographs of the steels after hot rolling and interrupted cooling: **a** CMn, **b** 1.7 Cu, **c** TiMo*, **d** 1.7 CuTiMo (*Reproduced with permission from Phaniraj et al. [12]. Copyright 2015 Elsevier)

**Fig. 2:**
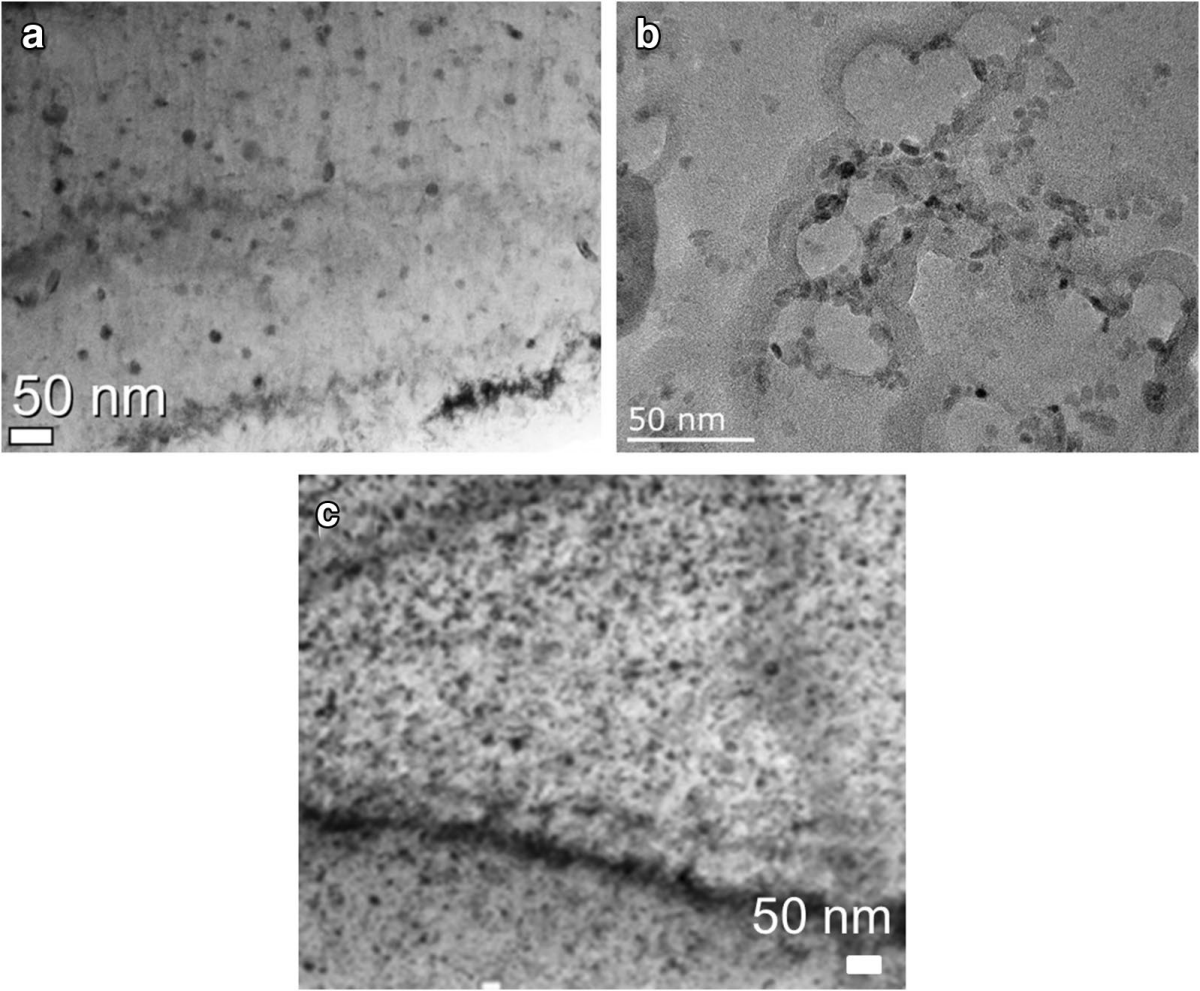
**a** Bright field image from 1.7 Cu foil showing copper precipitates. **b** Extracted carbide precipitates from TiMo*. **c** From 1.7 CuTiMo foil showing both carbide and Cu precipitates (*Reproduced with permission from Phaniraj et al. [12]. Copyright 2015 Elsevier)

The microstructure in all the steels consists mainly of polygonal ferrite (Fig. 1a–d) and some pearlite. The ferrite grain size of both CMn and 1.7 Cu in Fig. 1a, b is 16 μm whereas TiMo and 1.7 CuTiMo have relatively finer grain size of 12 μm as shown in Fig. 1c, d. The copper precipitates appear as circular shapes with darker contrast in the bright field image from 1.7 Cu (Fig. 2a). Image analysis showed that the size of copper precipitates was the range from 6 to 23 nm and the average size was about 9 nm. The precipitates having size less than ~3 nm size are fully coherent with the matrix and could not be observed in TEM because of poor diffraction contrast [12–14]. Figure 2b is a TEM micrograph of the carbide precipitates extracted from TiMo from the previous study [12]. The carbide precipitates had shapes with different aspect ratios ranging from spherical to cylindrical. The size of the precipitates was reported in a previous study by the present authors to be in the range from 2 to 8 nm and the average size is about 4 nm. It was confirmed, based on composition analysis and electron diffraction, that the precipitates were face center cubic (Ti,Mo)C carbides [12]. Figure 2c showed full of nano-precipitates in 1.7 CuTiMo and the presence of Cu and (Ti,Mo) precipitation in 1.7 CuTiMo was also confirmed by EDS spectrum analysis. However, the detailed information of the size and fraction of Cu and TiMoC was not able to be extracted.

### SANS data comparison

**Fig. 3:**
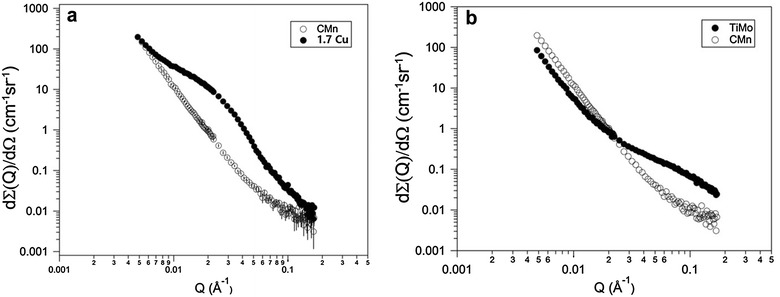
Macroscopic differential scattering cross section plotted against the scattering vector Q (Å^−1^). The scattering intensity of CMn steel compared with **a** 1.7 Cu and **b** TiMo

The macroscopic nuclear scattering cross section (henceforth referred to as intensity) as a function of the scattering vector is shown in Fig. 3. In order to discern the changes in the scattering curve after the alloying additions of Cu and Ti-Mo the respective scattering curves are plotted along with the scattering curve from CMn. While the CMn steel shows only a power law scattering, the 1.7 Cu steel (Fig. 3a) and the TiMo steel (Fig. 3b) show knee (i.e., Guinier-like scattering) around Q ~0.02 Å^−1^, and Q ~0.1 Å^−1^, respectively. The Guinier scattering could be a result of the precipitation of copper and (TiMo)C, which indicates the size of copper precipitate is larger than that of TiMo. All samples including CMn, 1.7 Cu, TiMo, and 1.7 CuTiMo that is present in the Additional file 1, show low angle upturns (i.e., power law scattering), which may be originated from the large inhomogeneity like the grain boundaries.

### SANS analysis

**Fig. 4:**
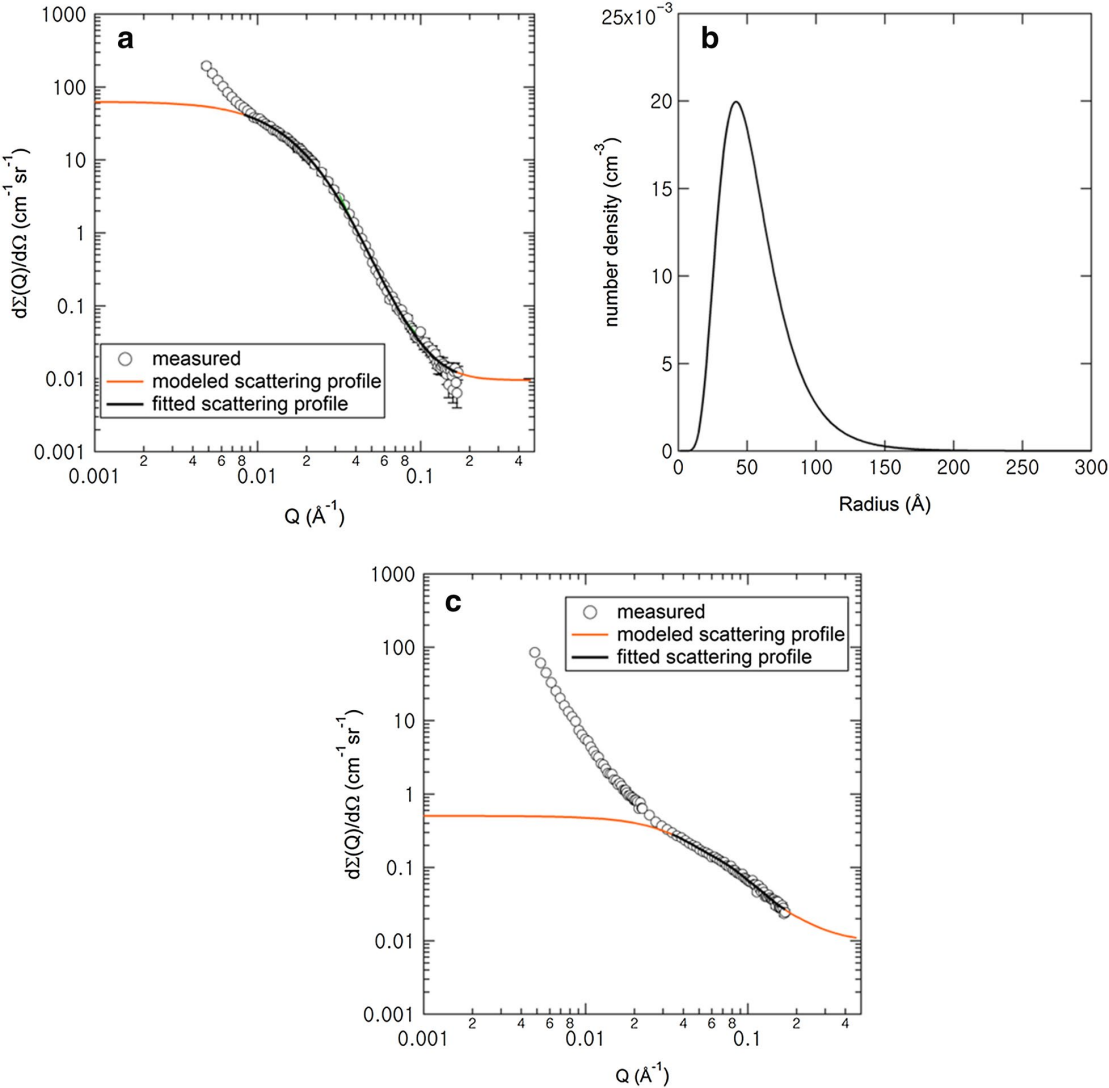
Macroscopic differential scattering cross section and modelled and fitted scattering profile for **a** 1.7 Cu and **c** TiMo. **b** Lognormal size distribution of copper precipitates calculated from the fitting

The copper precipitates were assumed to be spherical based on TEM observations and having a lognormal size distribution. The lognormal sphere model was chosen in the NIST SANS data analysis software [25, 26]. The model parameters used to fit the scattering profile are scattering length density (SLD), precipitate radius and standard deviation of distribution, volume fraction and background. The SLD of the ferrite matrix is calculated from ρ_Fe_ = N_Fe_b_Fe_ = 8.01 × 10^−6^ A^−2^, where N_Fe_ is the number density of iron atoms in ferrite and b_Fe_ is the coherent neutron scattering length of iron [27]. Similarly, the SLD for copper precipitates was calculated by assuming that the precipitates were pure copper (ρ_Cu_ = 6.53 × 10^−6^ A^−2^). The model was fitted to the nuclear scattering data by fixing the background (*dΣ*/*dΩ* = 0.01), and iterating other parameters with constraints. The measured, modelled and fitted scattering profiles are shown in Fig. 4a, and the corresponding lognormal distribution of the radius of copper precipitates is shown in Fig. 4b. The maximum size of copper precipitates (23 nm) measured from TEM micrographs is within the calculated radius range with non-negligible number density (i.e. radius up to ~150 Å). The calculated mean diameter of copper precipitates is 10 nm which is close to the measured mean value of 9 nm, and the calculated volume fraction is 9.5 × 10^−3^.

The scattering profile for TiMo could be best fitted by assuming that the carbide precipitates have the shape of an elliptic cylinder. The SLD for (Ti,Mo)C was estimated as ρ_TiMoC_ = N_TiMoC_(x_Ti_b_Ti_ + x_Mo_b_Mo_ + x_C_b_C_) = 3.92 × 10^−6^ A^−2^, where x is the mass fraction of the element. The model fitted in the Q-range from Q = 0.029 to 0.169 Å^−1^ shows the particle looks like an elliptic cylinder in shape. The calculated carbide particle dimensions were: major radius  = 2.37 nm, length = 13.83 nm and ellipticity ν = 6.17, and the calculated volume fraction is 7.6 × 10^−3^. It is difficult to directly compare with the measured dimensions of the carbide particles in 2D micrographs. However, the average diameter of the carbide particle matches well with the mean size measured from TEM micrographs. It should be noted that the SANS analysis of 1.7 CuTiMo steel was not able to given in this study because the existing SANS model was not optimal enough to interpret the system.

### Mechanical properties

The yield strength and tensile strength and % elongation for the steels from the tensile tests are given in Table 2. The yield strength increases by ~70% after alloying with copper and with further addition of Ti-Mo increases by ~185%.

**Table 2:** Measured yield strength (YS), tensile strength (TS) and % elongation, and precipitation strengthening contribution from Eq. 1. The data for CMn and TiMo are taken from our previous work [12]

Steel	YS (MPa)	TS (MPa)	% Elongation	σ_ppt_ (MPa)
CMn	267	401	33	–
1.7 Cu	452	567	15.2	158
TiMo	642	770	16.6	347
1.7 CuTiMo	695	840	11.6	380

We can estimate precipitation strengthening based on the conventional strengthening mechanisms as follows. The 1.7 Cu and 1.7 CuTiMo steels have base composition similar to CMn, and have a microstructure that is predominantly ferritic. Microstructure analysis showed that nanocrystalline precipitates form in the steels, and that the 1.7 CuTiMo steel has a relatively smaller grain size, thereby the contribution to yield strength from precipitates can be determined using Eq. 1

1 $$\sigma_{ppt} = \sigma_{YS} - \sigma_{YS}^{CMn} - \Delta \sigma_{g} - \sigma_{Cu}$$

where, Δσ_g_ = 21 MPa is the increment in the strength because of the slightly finer grain size of 1.7 CuTiMo, when compared with CMn steel, calculated using 17.402d^−1/2^ [3, 28] and σ_Cu_ is the contribution from solid solution strengthening by copper (38 MPa per wt% Cu [1]). The amount of copper remaining in solution after precipitation was calculated to be 0.7 wt% [12]; this amount was used to calculate σ_Cu_ in Eq. 1. The solubility of titanium and molybdenum in ferrite is low and is assumed to have negligible solid solution strengthening effect. The precipitation strengthening contribution calculated using Eq. 1 is given in Table 2. The increment in yield strength due to copper precipitation in 1.7 Cu is 158 MPa. This is comparable to the improvement in yield strength of 162 MPa reported in Fe–Cu alloy containing 10 nm size copper precipitates [29]. The precipitation strengthening contribution in the 1.7 CuTiMo steel is 380 MPa, which is nearly one and half times the yield strength of the CMn steel.

Now, the question is how this combined strengthening of Cu and (Ti,Mo)C precipitates can be estimated based on their individual strengthening response. There have been two major methods of summing the strengthening effects of multiple precipitates: Linear superposition and Pythagorean superposition [30]. The precipitation strengthening contribution due to (Ti,Mo)C carbide precipitates was determined using Eq. 1 to be 347 MPa in our previous work [12]. Using the linear superposition, the incremental increase in the yield stress of 1.7 CuTiMo is calculated to be 512 MPa which is much higher than the measured incremental increase of 1.7 CuTiMo in Table 2. The reason of this overestimation may be because the linear superposition is more applicable to the case of a small number of strong precipitates added in the matrix with a large number of weak precipitates [30]. By contrast, the Pythagorean superposition (Eq. 2) has been shown to give a good fit in other multiple precipitate strengthening when the increment in yield stress is considered in terms of the individual incremental increases in the yield stress.

2 $$\sigma_{ppt} = \sqrt {\sigma_{Cu\, ppt}^{2} + \sigma_{(Ti,Mo)C\, ppt}^{2} }$$

where *σ*_*ppt*_ is the increment in yield stress due to precipitation strengthening in 1.7 CuTiMo.

Using Eq. 2 gives the increment in strengthening in 1.7 CuTiMo to be 387.6 MPa which is in good agreement with the value derived from the measured yield strength viz. 380 MPa. Therefore, strengthening by two independent populations of nano-sized carbide and Cu precipitates can be well described by the Pythagorean superposition instead of the linear superposition.

The effectiveness of the Pythagorean superposition for dual nanoprecipitation strengthening is supported by our SANS measurement. The precipitation strengthening contribution can also be estimated directly from the Ashby–Orowan relationship (Eq. 3) [28] using the precipitate diameter and volume fraction.

3 $$\sigma_{ppt} = \frac{K}{d}f^{1/2} \ln \frac{d}{b}$$

where *K* is a constant = 5.9 N/m [3], *b* is the burgers vector (0.246 nm), *d* and *f* are the diameter and volume fraction of precipitates. The precipitation strengthening values calculated using Eq. 3 and the measured volume fraction and precipitate size from SANS analysis are given in Table 3. The precipitation strengthening contribution is 33% higher and 11% lower than the values determined using Eq. 1 for 1.7 Cu and TiMo respectively. Equation 3 is derived based on the assumption that the precipitates are incoherent, spherical and randomly dispersed on the slip plane. Thereby, given that the precipitate size calculated from SANS data are the average size over the sample volume and that the precipitate shape has different aspect ratios the difference between the estimated and measured values is reasonable. Above all, the increment in strengthening in 1.7 CuTiMo is calculated to be 380 MPa using Eq. 2, which is consistent with the value derived from the measured yield strength viz. 380 MPa. Therefore, the Ashby–Orowan relationship with Pythagorean superposition can be a good estimate for the dual precipitation strengthening of nano-sized carbides and Cu precipitates which are well dispersed in ferritic steels.

**Table 3:** Precipitate size, volume fraction and precipitation strengthening contribution σ_ppt_ from Eq. 3

	Size (nm)		SANS vol. fraction	Calculated σ_ppt_ (MPa)
	SANS	TEM		
1.7 Cu	10.12	9	9.3986 × 10^−3^	210.08
TiMo	4.74^a^	4	7.4509 × 10^−3^	317.84

^a^Major diameter

## Conclusions

Three low carbon steels: plain carbon steel, alloyed with Cu and with Cu, Ti, and Mo were prepared and subjected to hot rolling followed by interrupted cooling. The microstructure was characterized using SEM, TEM and SANS. The specimens were tested in tension to determine mechanical properties. Precipitation strengthening contributions were calculated and compared with measured values.

1. Alloying with Cu, Ti and Mo increased the yield and tensile strength of the hot rolled and interrupt cooled low carbon steel from 267 to 695 MPa and 401 to 840 MPa.
2. TEM and SANS analysis show that mean size of copper precipitates of size is ~10 nm.
3. The increment in yield strength due to precipitation strengthening in 1.65Cu steel is 158 MPa. The precipitation strengthening contribution of copper and carbide precipitates jointly, in the 1.7 CuTiMo steel, is 380 MPa.
4. The Pythagorean superposition gives a good estimate of the combined increment in precipitation strengthening due to (Ti,Mo)C carbides and copper precipitates.
5. The precipitation strengthening contribution calculated using the Ashby–Orowan equation with volume fraction and size from SANS analysis is in reasonable agreement with the measured values.

## Additional file

**Additional file 1.** Macroscopic nuclear scattering cross section as a function of the scattering vector for all four samples.
