# Erratum to: Understanding dual precipitation strengthening in ultra-high strength low carbon steel containing nano-sized copper precipitates and carbides

**DOI:** 10.1186/s40580-017-0114-1

**Published:** 2017-07-27

**Authors:** M. P. Phaniraj, Young-Min Shin, Woo-Sang Jung, Man-Ho Kim, In-Suk Choi

**Affiliations:** 1 High Temperature Energy Materials Research Center, Korea Institute of Science and Technology, Seoul, 136-791 Republic of Korea; 2 Advanced Analysis Center, Korea Institute of Science and Technology, Seoul, 136-791 Republic of Korea

## Erratum to: Nano Convergence (2017) 4:16 DOI 10.1186/s40580-017-0110-5

After publication of the original article [1], the authors noticed one error with the following sentence in “Conclusion” section:

“3. The increment in yield strength due to precipitation strengthening in 1.65Cu steel is 158 MPa.”

“1.65Cu” should be corrected to “1.7Cu”.

The original article has been updated.
