# Supplementary material for: Understanding dual precipitation strengthening in ultra-high strength low carbon steel containing nano-sized copper precipitates and carbides
Source: Nano Converg. 2017 Jul 3;4:16. doi: 10.1186/s40580-017-0110-5 (PMC5494287; doi:10.1186/s40580-017-0110-5)
Supplement: Supplementary file 1 — Additional file 1. Macroscopic nuclear scattering cross section as a function of the scattering vector for all four samples. [file 40580_2017_110_MOESM1_ESM.docx]

**Supporting Information**

**Understanding dual precipitation strengthening in ultra-high strength low carbon steel containing nano-sized copper precipitates and carbides**

M. P. Phaniraj^a+^, Young-Min Shin^a+^, Woo-Sang Jung^a^, Man-Ho Kim^b*^, In-Suk Choi^a,^*

^a^High Temperature Energy Materials Research Center, Korea Institute of Science and

Technology, Seoul 136-791, Republic Korea

^b^Advanced Analysis Center, Korea Institute of Science and Technology, Seoul 136-791, Republic Korea

* Corresponding authors: [insukchoi@kist.re.kr](mailto:insukchoi@kist.re.kr), [manhokim@kist.re.kr](mailto:manhokim@kist.re.kr)

+The authors contributed equally to this work.

The macroscopic nuclear scattering cross section (henceforth referred to as intensity) as a function of the scattering vector for CMn, 1.7Cu, TiMo, CuTiMo, 1.7CuTiMo is shown in Fig.S1. In order to discern the changes in the scattering curve after the alloying additions of Cu and Ti-Mo the respective scattering curves are plotted along with the scattering curve from CMn. SANS measurement for CuTiMo and 1.7CuTiMo steel also shows clear difference of SANS data compared to CMn. The curve follows that of 1.7 Cu at low Q intensity while it follows the TiMo curve at high Q, which indicates that both Cu and (TiMo)C are well dispersed in the steel matrix. The SANS analysis for extracting the volume fraction and precipitate size were not able to present here since multiple precipitates of similar size in the steel matrix are difficult to analyze by SANS analysis because the existing SANS model was not optimal enough to interpret the system.


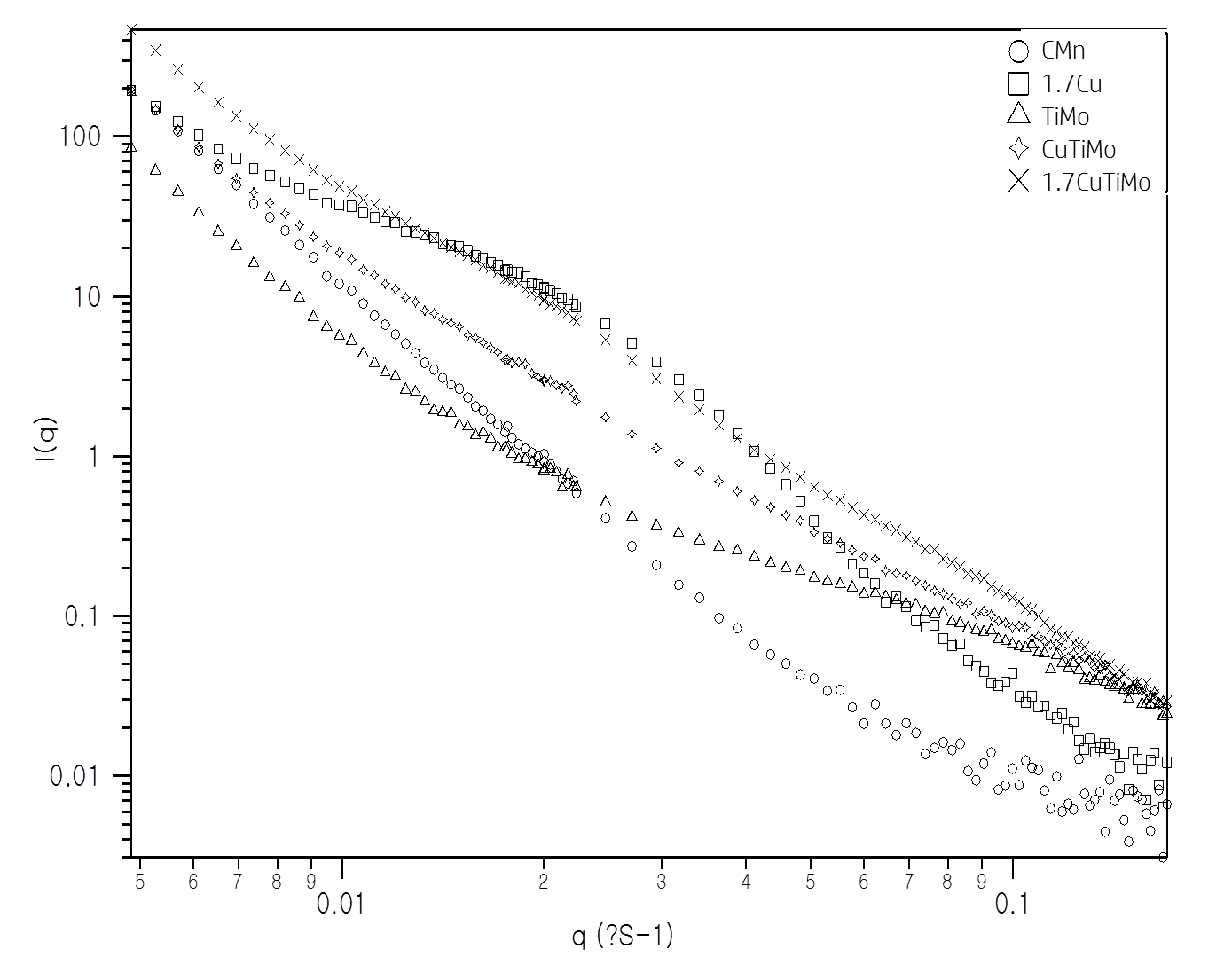


Figure S1 Macroscopic nuclear scattering cross section as a function of the scattering vector for CMn, 1.7Cu, TiMo, CuTiMo, 1.7CuTiMo
